# A comparative plastomic analysis of *Ziziphus jujuba* var. *spinosa* (Bunge) Hu ex H. F. Chow and implication of the origin of Chinese jujube

**DOI:** 10.1093/aobpla/plad006

**Published:** 2023-02-21

**Authors:** Shuhui Du, Xiaoyan Hu, Yuanting Guo, Shengji Wang, Xiuyun Yang, Zhenzhen Wu, Yuyin Huang

**Affiliations:** College of Forestry, Shanxi Agricultural University, Taigu, Shanxi, China; College of Food Science and Technology, Shanxi Agricultural University, Taigu, Shanxi, China; College of Forestry, Shanxi Agricultural University, Taigu, Shanxi, China; College of Forestry, Shanxi Agricultural University, Taigu, Shanxi, China; College of Forestry, Shanxi Agricultural University, Taigu, Shanxi, China; Taian Dushihuaxiang Agricultural Technology Co., Ltd, Taian, Shandong, China; Shandong Huinongtianxia Science and Technology Information Consulting Co., Ltd, Taian, Shandong, China

**Keywords:** *Ziziphus jujuba* var, *spinosa*, plastomic variation, SNPs, phylogenomic analysis

## Abstract

Comparative plastomics can be used to explicitly dissect various types of plastome variation. In the present study, the plastome variation pattern of *Ziziphus jujuba* var. *spinosa* (also called sour jujube) and its phylogenomic relationship with Chinese jujube were investigated. Plastomes of 21 sour jujube individuals were sequenced and assembled. The length of the sour jujube plastomes ranged between 159399 and 161279 bp. The plastomes exhibited collinearity of structure, gene order and content. The most divergent regions were located in the intergenic spacers, such as *trnR-UCU*-*atpA* and *psbZ*-*trnG-UCC*. Sliding window analysis demonstrated that the sequence variation among the sour jujube plastomes was relatively low. Sixty-two to 76 SSRs with 4 motif types were identified in the sour jujube plastomes with a predominant motif type of A/T. Three protein-coding genes exhibited higher nonsynonymous/synonymous substitution ratios, indicating that these genes may undergo positive selection. A total of 80 SNPs were detected and 1266 potential RNA editing sites of 23 protein-coding genes were predicted. In the phylogenomic tree constructed, sour jujube has a sister relationship to Chinese jujube, which indicates that Chinese jujube may have originated or been domesticated from sour jujube. The present study explicitly investigated the individual-level plastome variation of sour jujube and provides potential valuable molecular markers for future genetic-related study of this lineage.

## 1 Introduction

The chloroplast is a plant organelle originating from a primitive cyanobacterium and performs a fundamental role in plant photosynthesis and other functions (Gray and Doolittle 1982; Timmis *et al*. 2004; Zhou *et al*. 2021). The structure and gene content of the chloroplast genome (plastome) are generally conserved among photosynthetic land plant organisms. It usually contains a large and a small single copy region (LSC and SSC), which are separated by two inverted repeat sequences (IRs) (Palmer 1991). The conserved nature of the plant plastome makes it homogeneous enough to allow comparative studies across high level taxa, and it is also sufficiently divergent to capture various evolutionary events within a specific species (Zhou *et al*. 2021). Comparative plastomic analyses among closely related species or individuals of the same species can provide a more exhaustive understanding of the evolutionary trajectory of these organisms (Shaw *et al*. 2005; Gao *et al*. 2018; Liu *et al*. 2021). Furthermore, in contrast to the nuclear genome, which is bi-parentally inherited, the plastome is uniparentally inherited without recombination in most flowering plants (Nock *et al*. 2019). Consequently, chloroplast data has been widely utilized in research on the evolutionary history of various plant species (Du *et al*. 2015; Wang *et al*. 2013; Ren *et al*. 2020).

Understanding the relationship between closely related domesticated and wild germplasm is important to guide the introduction of novel genetic variation into selective breeding, and to prioritize strategies to conserve novel wild germplasm (Brozynska *et al*. 2016; Luo *et al*. 2017; Nock *et al*. 2019). Most major modern crops are derived from Northern Hemisphere Monocotyledon and/or core Eudicotyledon species that were domesticated thousands of years ago (Miller and Gross 2011). *Ziziphus jujuba* var. *spinosa*, also known as sour jujube, is a deciduous shrub plant species belonging to the Rhamnaceae and is widely distributed in Northern China (Zhang *et al*. 2015). It has significant ecological value and is typically used for soil and water conservation (Wang *et al*. 2018). Given the fruit’s nutritional value and the kernel’s medicinal importance, it has been economically important in China for more than 2000 years (Qu 1982). In the Flora of China, *Z. jujuba* var. *spinosa* is classified as a *varietas* of *Z. jujuba* (Chinese jujube). However, based on its morphology and other evidence, some researchers have suggested that sour jujube should be classified as an original lineage and Chinese jujube might have originated or been domesticated from sour jujube (Peng 1991), and that the evolutionary path may involve several different patterns (Liu 1993). But until now no molecular evidence has been pinpointed to support this hypothesis, except that Huang *et al*. (2015) proposed an independent origin and domestication route for Chinese jujube using cpSSR markers. Also, others have suggested that sour jujube and Chinese jujube are two different species and that the scientific name of sour jujube is *Ziziphus acidojujuba* (Huang *et al*. 2015; Zhang *et al*. 2015; Huang *et al*. 2017; Guo *et al*. 2020).

Previous research on sour jujube mainly focused on nutritional and medicinal ingredients, responses to various abiotic stresses, as well as genetic diversity and structure (Kang *et al*. 2008; Zhang *et al*. 2015; Li *et al*. 2017; Yan *et al*. 2017; Hu *et al*. 2021). However, some other basic issues concerning sour jujube based on chloroplast datasets, such as phylogeography and population genetic research, have rarely been addressed. Zhang *et al*. (2015) investigated the genetic diversity and structure of sour jujube, but only nSSR data were used. In an attempt to guide future chloroplast-based studies, plastomes of individuals from different populations across the distribution range of sour jujube were sequenced and analyzed in the present study. Furthermore, phylogenomic analysis based on plastomes of species from Rhamnaceae and closely related families was conducted to verify the phylogenetic relationship between sour jujube and Chinese jujube.

## Materials and methods

### Plant material and DNA extraction

**Figure 1.**
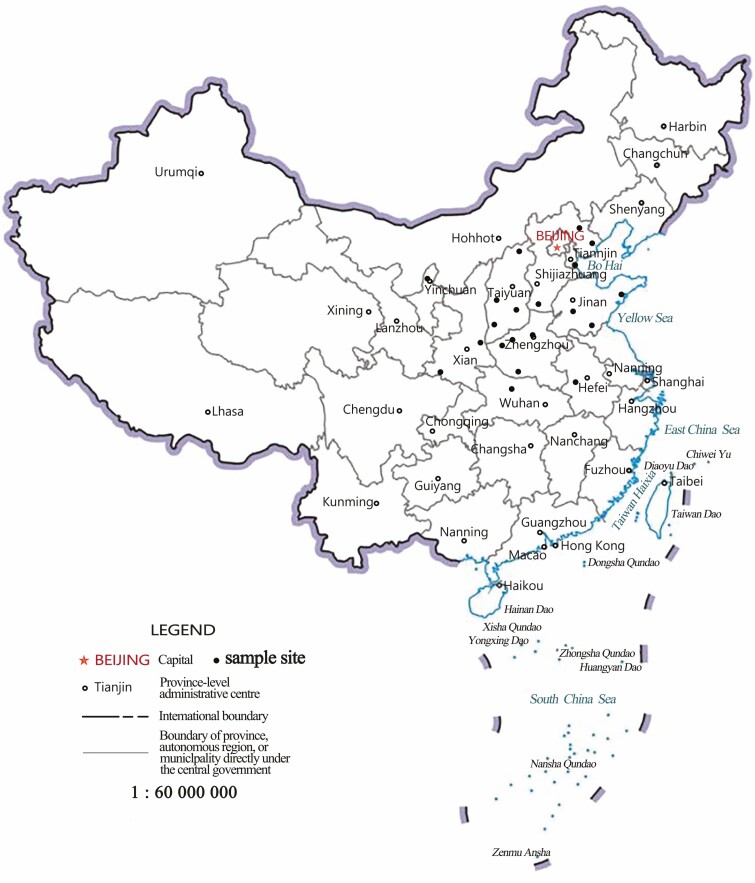
Individuals collected in this study. Black dots indicate the location of the sampled populations.

Twenty-one sour jujube individuals sampled from 21 natural populations in China were utilized in this study (Fig. 1, [see Supporting Information—Table S1]). Fresh leaves were collected and stored at room temperature in silica gel prior to DNA extraction. Total genomic DNA was extracted using a DNeasy Mini Kit (Tiangen, China) according to the manufacturer’s protocols. DNA concentration was quantified using a Nanodrop 2000 and the size and quality of the DNA were visualized on a 1.0% agarose gel.

### Library construction and sequencing

Genomic DNA was normalized to 50 ng/ml for library construction. Sequence libraries for each individual were prepared using an Illumina Nextera XT DNA Library Preparation Kit according to the manufacturer’s instructions (Illumina, USA). Sequence libraries were quantified using a Bioanalyzer 2100 (Agilent, USA). Each sample was barcoded with a unique index and libraries were pooled, and whole genome sequence data were generated using an Illumina HiSeq 2500 platform.

### Genome assembly, annotation and sequence feature

Using a previously reported plastome of *Z. jujuba* var. *spinosa* (KX266830) as a reference, the plastomes of 21 individuals sampled in this study were assembled using NOVOPlasty 3.094 (Dierckxsens *et al*. 2017) with default parameters. The assemblies were annotated via GeSeq with default parameters (Tillich *et al*. 2017) along with 3rd party tRNA annotation using ARAGORN 1.2.38 (Laslett *et al*. 2004) and tRNAscan-SE 2.0.7 (Chan *et al*. 2021). The annotations were manually examined and revised by comparison with homologous genes and reference plastomes using Geneious 10.2.2 (https://www.geneious.com). The circular plastomic maps of the 21 individuals were drawn using OGDRAW with default values (Stephan *et al*. 2019). Consensus sequences were extracted for each sample and aligned using MAFFT with default settings (Katoh *et al*. 2017). The 21 plastomes were compared and overall divergence was analyzed in mVISTA (Frazer *et al*. 2004) with Shuffle-LAGAN mode, and the previously published sour jujube plastome (KX266830) was used as the basis for comparisons. The borders of four different regions within the 21 plastomes and another six *Ziziphus* plastomes used in the following phylogenomic analysis were visualized using IRscope (Ali *et al*. 2018). The nucleotide diversity value (Pi) was calculated by DnaSP 6 (Librado and Rozas 2009) using a sliding window length of 600 bp and a 200 bp step size.

### Repeat sequence identification

Repeat elements in the 21 sour jujube plastomes were investigated using different methods. The position and type of SSRs were screened using the microsatellite identification tool MISA-web (Thiel *et al*. 2003; Sebastian *et al*. 2017). SSRs were identified with thresholds of 10, 6, 5, 5, 5 and 5 repeat units for mono-, di-, tri-, tetra-, penta- and hexa-nucleotides, respectively. Forward, reverse, complement and palindromic repeats were examined via REPuter (Kurtz and Schleiermacher 1999) with parameter settings as follows: hamming distance was 3, minimum and maximum sizes of repeats were 30 and 500 bp, and redundant repeats were manually removed (Yuan *et al*. 2021). RNA editing sites within each protein coding gene of the 21 sour jujube plastomes were predicted through PREP-Cp (Mower 2009) with a cutoff value of 0.8.

### Synonymous (Ks) and nonsynonymous (Ka) substitution rate analyses

To evaluate the selection pressure that sour jujube chloroplast genes may have experienced, 73 homologous plastome protein-coding genes were extracted from all *Ziziphus* species used in this study and aligned separately using Geneious 10.2.2. Alignments for *rpl22* and *ndhF* were truncated at frame-shift mutations close to their 3ʹ ends. The alignment file was then exported from Geneious and analyzed in R using SeqinR (Charif and Lobry 2007) to calculate pairwise Ka and Ks values. The Ka/Ks ratio was then calculated from the mean Ka and Ks values averaged over all pairwise comparisons.

### Phylogenomic analysis

To research the phylogenetic relationships and allow for comparisons among different Rhamnaceae species, phylogenomic trees were constructed using MrBayes 3.2 (Ronquist *et al*. 2012), Raxml-NG (Kozlov *et al*. 2019) and RaxmlGUI 2.0 (Stamatakis 2014; Edler *et al*. 2021) with species from four tribes of Rhamnaceae, namely Zizipheae, Ventilagineae, Pomaderrea and Rhamneae, as well as species from Barbeyaceae, Elaeagnaceae and Moraceae (*Barbeya oleoides*, *Elaeagnus macrophylla*, *Hippophae rhamnoides*, *Morus alba* and *Cannabis sativa*) as outgroups based on the 73 common protein coding sequences extracted by Geneious 10.2.2 and aligned with MAFFT. All the other species’ plastomes were directly downloaded from NCBI ([see Supporting Information—Table S2]). A GTR+GAMMA+I nucleotide substitution model was determined by JModeltest 3.7 (Posada 2008) for the dataset. For Bayesian Inference of phylogeny with MrBayes, two independent runs of Metropolis-coupled MCMC were conducted simultaneously, with each run being one cold chain and three incrementally heated chains and all started randomly in the parameters space. All other parameters were set to default. Twenty million generations were run and trees were sampled once every 1000 generations. The program Tracer 1.5 was used to check for stationarity. The first 25% of sampled trees were discarded as burn-in and the posterior probabilities (PP) were calculated from the remaining trees. For maximum likelihood tree construction in Raxml, 1000 bootstrap replicates were conducted. Figtree 1.4.4 was utilized to visualize the result trees.

## Results

### Genome assembly, organization, gene content and features

**Figure 2.**
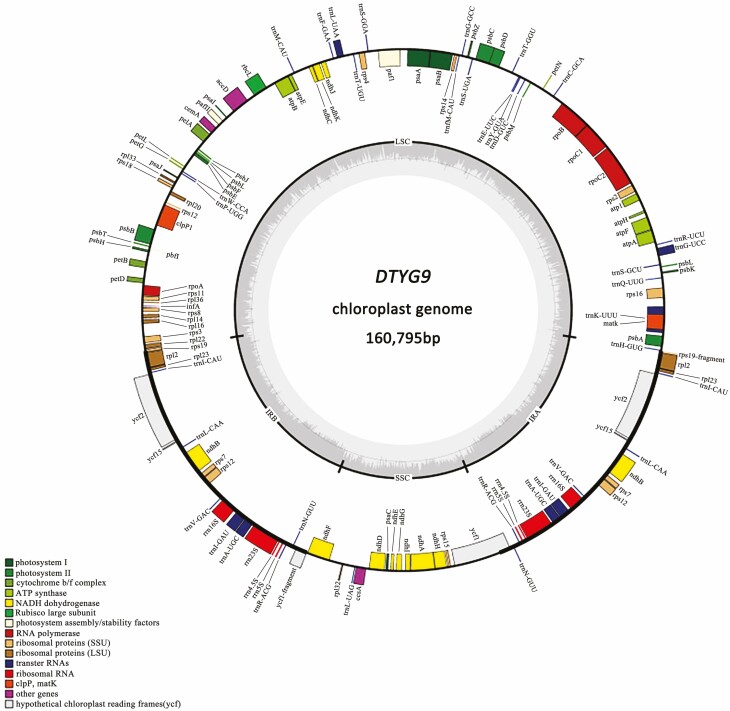
The circular plastome map of sour jujube with gene locations on both strands, along with an indication of the IR, LSC and SSC regions. Predicted functional genes belonging to different categories were color-coded.

Illumina pair-end sequencing produced a total of 30.618 Gb clean data for 21 sour jujube individuals. The average N50 and N90 contig lengths of the 21 sour jujube individuals were 99623 and 42485 bp, respectively. The length of the newly assembled sour jujube plastomes ranged from 159399 to 161279 bp. GC content varied between 36.51% and 37.30% with an average value of 36.84%. Detailed information about the newly assembled plastomes is listed in Table 1. The complete sour jujube plastomes displayed a circular DNA molecule with a typical quadripartite structure with IRa and IRb separated by LSC and SSC (Fig. 2). The plastomes exhibited collinearity of gene order and content, which encoded an identical set of 135 predicted functional genes containing 88 protein-coding genes, 39 tRNA genes and 8 rRNA genes. Within these predicted functional genes, 20 were duplicated in the IR regions of the plastome. Furthermore, 15 distinctive genes, such as *atpF*, *ndhA*, *ndhB*, *rpl2*, *trnA-UGC* and *trnC-ACA*, contained one single intron, while *clpP* and *pafI* contained two introns ([see Supporting Information—Table S3]).

**Table 1.** Detailed information about the plastomes of 21 sour jujube individuals.

Individual	Length (bp)	GC content (%)	Assembly coverage	LSC (bp)/GC(%)	SSC (bp)/GC(%)	IR (bp)/GC(%)
HBCD19	160876	36.92	99.16%	88526/34.7	19392/30.9	26479/42.6
HBQHD5	160730	36.69	99.07%	88690/34.5	19364/30.8	26338/42.7
HBXT5	160691	36.86	99.05%	88411/34.7	19358/30.9	26461/42.7
CZCZ1	160750	36.86	99.09%	88378/34.7	19378/30.9	26497/42.6
DTYG9	160795	36.84	99.11%	88481/34.7	19356/30.8	26479/42.6
JZTG11	160764	36.69	99.10%	88737/34.5	19353/30.8	26337/42.7
LLXY7	159754	36.89	98.47%	87701/34.8	19107/30.8	26470/42.9
YCRC14	161259	36.74	99.40%	88945/34.5	19356/30.9	26479/42.6
SDLS10	161211	36.75	99.37%	88897/34.6	19356/30.9	26478/42.6
LYMY19	160705	36.70	99.06%	88674/34.5	19359/32.4	26336/43.0
SDTA10	161278	36.77	99.41%	88964/34.6	19356/30.9	26479/42.6
TJWQ2	161100	36.98	99.30%	88862/34.6	19366/30.9	26436/42.6
NXYC4	161062	36.80	99.28%	88746/34.6	19358/30.9	26479/42.6
SXHZ9	161279	36.76	99.41%	88888/34.6	19361/30.9	26515/42.6
SXWN9	160918	36.79	99.19%	88640/34.6	19356/30.9	26461/42.7
HNNY2	159994	36.99	98.62%	87751/34.9	19259/31.0	26492/42.6
HNSMX5	160347	36.93	98.84%	87995/34.8	19368/30.9	26492/42.6
HNLY9	159399	37.30	98.25%	87244/35.5	19127/31.1	26514/42.6
HNZZ14	161100	37.01	99.30%	88862/34.6	19361/30.9	26436/42.6
AHLA1	160542	36.49	98.96%	88573/34.2	19339/30.5	26378/42.7
HBXY14	161080	36.85	99.29%	88751/34.6	19361/30.9	26484/42.7

### Structural comparison of sour jujube plastomes

**Figure 3.**
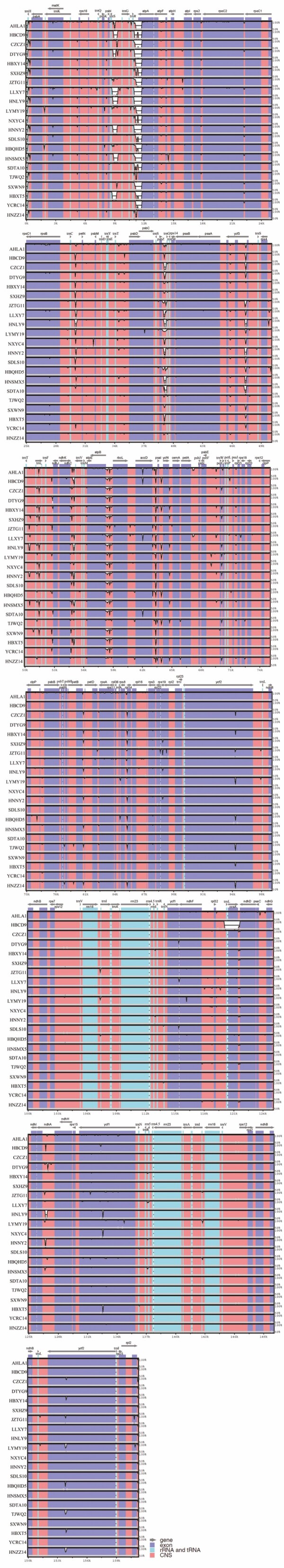
Visualization of the alignment of the sour jujube plastomes with a previously reported sequence as the reference. Gray arrows above the alignment indicate genes, including their orientation and position. The vertical scale represents the percentage of identity, ranging from 50 to 100%. Genome regions are color-coded as protein-coding genes, tRNAs, rRNAs and conserved noncoding sequences (CNSs).

The multiple sequences alignment performed in mVISTA with a previously reported plastome of *Z. jujuba* var. *spinosa* as a reference revealed the similarity of the plastomes of the 21 sour jujube individuals (Fig. 3). As expected, the IR regions showed lower sequence divergence than the SC regions. Non-coding regions exhibited higher sequence variation than the coding counterparts, and the most divergent regions were located in the intergenic spacers, such as *trnR-UCU*-*atpA* and *psbZ*-*trnG-UCC*. The most divergent coding regions included *matK*, *accD* and *ycf2* (these above mentioned regions were verified based on PCR amplification and Sanger sequencing, [see Supporting Information—Table S4]). Accordingly, these regions could aid in the future phylogenetic, phylogeographic and population genetic analysis of sour jujube. Obvious large insertions/deletions (indels) were present in the intergenic spacers of the LSC and SSC regions of some individuals, such as *trnR-UCU*-*atpA* and *trnL-UAG*-*ndhD*.

**Figure 4.**
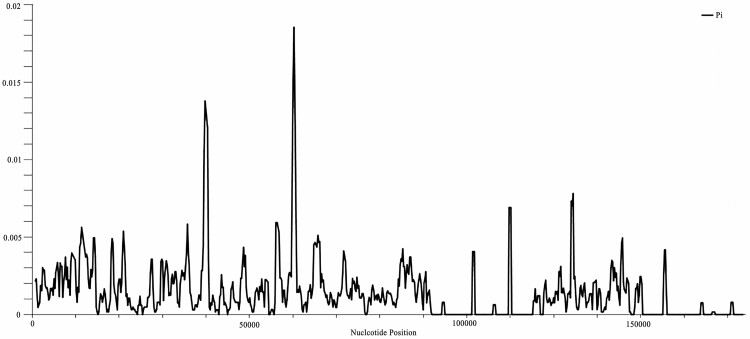
Result of the nucleotide variation (Pi) among the 21 sour jujube plastomes.

To detect mutational hotspots in the plastomes of the 21 sour jujube individuals, sliding window analysis was performed on the whole-plastome alignments. The results showed that the sequence variation among the sour jujube plastomes was relatively low, with Pi ranging from 0 to 0.01857, with an average value of 0.000132 (Fig. 4). Higher divergence in SC and lower divergence in IR regions were revealed, implying general conservatism of IR regions compared to other parts, which was consistent with characteristics of other angiosperm species’ plastomes (Yuan *et al*. 2021). Furthermore, the most divergent loci were located at *atpB*-*rbcL*, together with some other hypervariable regions, including *psaZ*-*trnG*, providing potential valuable markers for future genetic-related study of *Z. jujuba* var. *spinosa*.

### IR contraction and expansion

**Figure 5.**
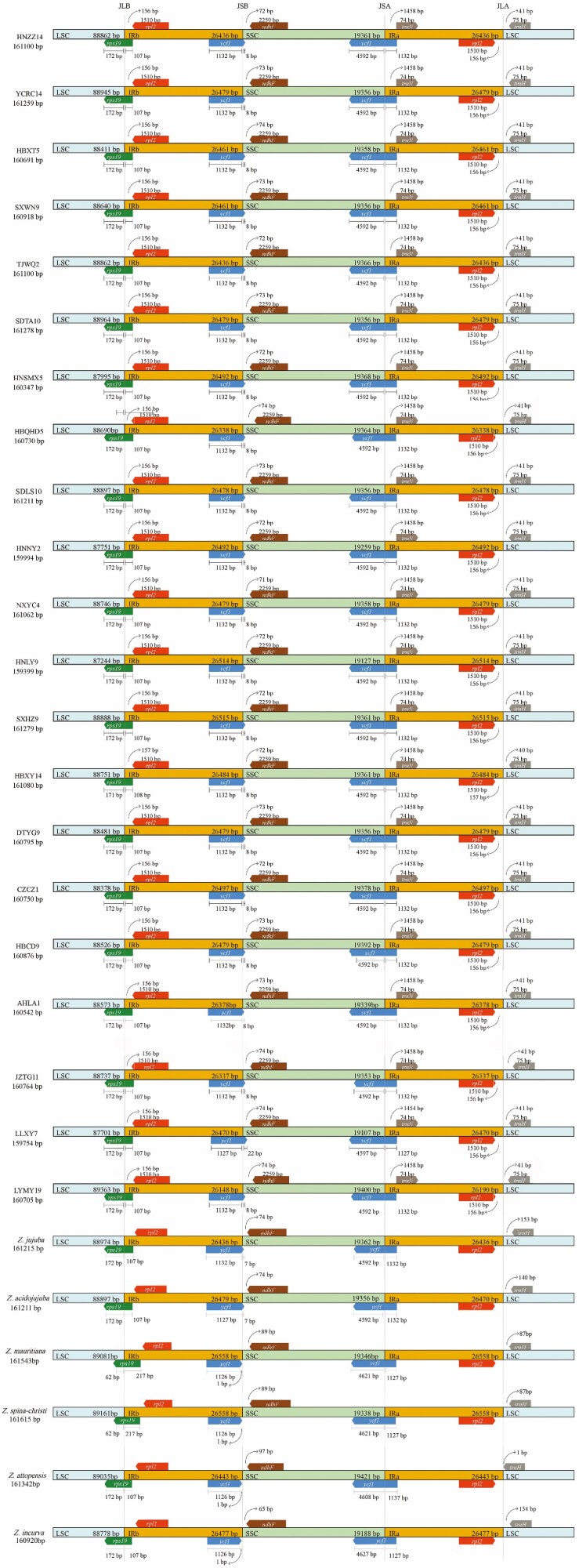
Comparison of the border positions of LSC, SSC, and IR regions among the 21 sour jujube plastomes.

The evolutionary trajectories of the contraction and expansion of IRs within sour jujube plastomes and other *Ziziphus* species were investigated and relatively conserved IR boundary fluctuations were demonstrated (Figure 5). The border of SSC/IRa (JSA) crossed by *ycf1* maintained a highly conserved state in *Ziziphus* plastomes. Symmetrically, the border of SSC/IRb (JSB) was located at another copy of *ycf1* and was adjacent to *ndhF*. A little inconsistent deviation of JSB from *ndhF* was observed. The junction of LSC/IRb (JLB) spanned *rps19* in all *Ziziphus* plastomes, and was proposed to be a putative ancestral JLB state in angiosperm species (Yuan *et al*. 2021). The boundary of LSC/IRa (JLA) was located in the region *rpl2*-*trnH*, and this was conserved in all analyzed *Ziziphus* plastomes.

### Repeat structure and SSRs analysis

Sixty-two SSR loci in individual HNNY2 to 76 SSR loci in individual JZTG11 with four motif types were identified in 21 sour jujube plastomes [see Supporting Information—Table S5]. Among the SSRs, 14 showed no variation among all the sour jujube individuals; detailed information is presented in Supporting Information—Table S1. SSR motifs presenting a heterogeneity frequency were predominantly rich in A/T bases (from 61 loci in individual AHLA1 to 67 loci in individuals LYMY19 and NXYC4), which accounted for nearly 92.74% of all the SSRs. The sour jujube plastomes exhibited similar SSR distribution patterns; SSRs were much more enriched in the LSC region (69.84–85.19%) than in the IR (7.94–9.16%) and SSC regions (6.35–8.15%). Of these SSRs, none were detected in protein-coding regions. On average, 74 long repeats were detected across the 21 sour jujube plastomes [see Supporting Information—Table S7]. These four kinds of long repeats (forward, reverse, complementary and palindromic repeats) displayed a constant pattern across all the sour jujube plastomes, as the forward and palindromic repeats were much more significantly enriched than the other two types. Moreover, it was found that many repeats (~61.54%) were located in the adjacent regions of IR boundaries, which were likely to lead to the IR boundary variation (Perry and Wolfe 2002; Yuan *et al*. 2021). Furthermore, most short repeats were located in the SC regions, for example, in individual AHLA1, ~80% 30 bp length repeats were detected in the LSC region.

### Characterization of substitution rates

We estimated pairwise Ka and Ks values for 73 common protein-coding genes in all the *Ziziphus* species’ plastomes used in this study. In most cases, synonymous nucleotide substitutions have occurred at a much higher rate than nonsynonymous nucleotide substitutions. Three protein-coding genes, including *atpE*, *rpl16* and *rps3*, exhibited a higher value of Ka than that of Ks, indicating that these genes may undergo positive selection during sour jujube plastome evolution [see Supporting Information—Table S8].

### SNP detection

A total of 80 SNPs (excluding SNPs with gaps) were detected throughout the 21 aligned sour jujube plastomes (some of these SNPs were verified based on PCR amplification and Sanger sequencing, [see Supporting Information—Table S4]). The ratio, less than 5%, was relatively small compared to the full length of the plastomes. Within the SNPs, 3 were detected in introns, 45 in noncoding sequences and 32 in functional genes [see Supporting Information—Table S9].

### RNA editing

A total of 1266 potential RNA editing sites of 23 protein-coding genes were predicted [see Supporting Information—Table S10]. In all 21 sour jujube plastomes, the event of Ser converting to Leu occurred with predominant frequency, accounting for 34.44% of all the RNA editing events, which was consistent with a previous study that found that the change from Ser to Leu became more frequent as the number of amino acids increases (Yuan *et al*. 2021). In contrast, Arg converting to Cys occurred with the lowest frequency. *Rps2* showed the highest frequency with 366 RNA editing events throughout the 21 sour jujube plastomes.

### Phylogenomic analysis

**Figure 6.**
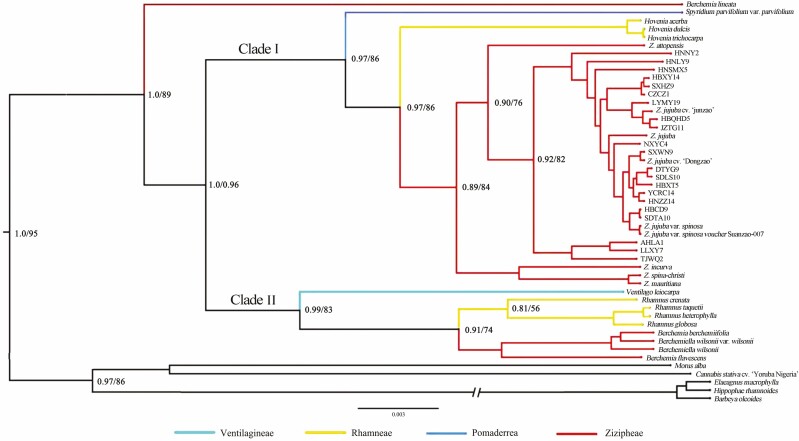
Phylogeny of Rhamnaceae reconstructed using 73 common protein-coding sequences.

The phylogenomic trees constructed using 73 common protein-coding sequences with MrBayes and Raxml were similar to each other with only a few differences in the PPs or the bootstrap values in some terminal nodes (Figure 6). All the outgroup species clustered in a separate clade with high PPs or bootstrap support values. In the clade comprising Rhamnaceae species, *Berchemia lineata* was separated from other *Berchemia*, and *Berchemiella* species were clustered in a single clade. We rechecked the protein-coding sequences of *B. lineata* and found that some of its nucleotide composition was unique and differed from all the other Rhamnaceae species used in the phylogenomic analysis. This may be the cause of the special position of *B. lineata* in the phylogenomic tree. All the remaining Rhamnaceae species were divided into two clades. Clade I consisted of species from Ziziphoids, and *Hovenia* showed a closer relationship to *Ziziphus* than to *Spyridium*. Furthermore, within clade I, all sour jujube and Chinese jujube individuals formed a single cluster. Clade II comprised four genera. *Ventilago* was more ancestral than the other three genera and *Rhamnus* showed a close relationship to *Berchemia* and *Berchemiella*. This phylogenetic pattern of species in Rhamnaceae was consistent with previous phylogenetic research using chloroplast fragments *rbcL* and *trnL-F* (Richardson *et al*. 2000b).

## Discussion

In this study, the complete plastome of 21 *Z. jujuba* var. *spinosa* individuals sampled across the distribution range in China was generated from high-throughput sequencing. The observed plastomic size of 159399–161279 bp was within the size range known for most land plants. They exhibited a typical quadripartite structure and contained a total of 135 predicted functional genes with highly conserved gene order, content and orientation. The newly assembled sour jujube plastomes showed some differences from previously published sour jujube plastomes in gene content (Huang *et al*. 2017). This may be the result of the differences in sequencing and assembly strategies of various studies. The presence of four rRNA genes clustered together in IR regions was identical to that reported in a wide range of taxa and other *Ziziphus* species (Yu *et al*. 2019; Liu *et al*. 2021; Yuan *et al*. 2021). In traditional molecular systematic and population genetic studies, some chloroplast fragments such as *matK*, *rbcL* and *trnL-trnF* have been recognized as among the best markers for barcoding and phylogeny reconstruction, due to their high level of divergence among different lineages (Hamzeh and Dayanandan 2004; Wang *et al*. 2012; Du *et al*. 2015; Du *et al*. 2020). Now that complete plastomic sequences can be easily sequenced and assembled, comparative plastomic and phylogenomic studies have grown substantially (Zhang *et al*. 2018; Liu *et al*. 2021; Mishra *et al*. 2021), which increases our knowledge about the evolutionary dynamics and variation of plant plastomes.

Whole-plastome alignment can explicitly elucidate the level of sequence divergence and easily identify large indels, which are extremely useful for future phylogenetic analyses and plant identification. In the present study, the mVISTA results showed that the IR regions presented lower sequence divergence than the SC regions, which was considered to result from copy correction between IR sequences and the elimination of deleterious mutations by gene conversion (Khakhlova and Bock 2006). Moreover, sequence differences among the sour jujube plastomes were more evident in the intergenic spacers and/or non-coding sequences, suggesting greater conservation in protein-coding regions. Although higher nucleotide variation (Pi) was present in certain divergence hotspot regions of the 21 sour jujube plastomes, the entire nucleotide variation exhibited a conserved tendency. All the above results illustrate the conservative nature of plant plastomes, especially in lower taxonomy units, like the population level of the present study. Sometimes even in higher taxonomy units, the plastomes of two species were completely identical (Yuan *et al*. 2021). Overall, a moderate divergence among specific regions was revealed, which has been demonstrated to be useful in phylogenetic studies of many families, even in Rhamnaceae (Anderson *et al*. 2005; Richardson *et al*. 2000b; Xiang *et al*. 2000). Even though relatively high nucleotide diversity was also detected in *rps14* and *psaC-ndhG*, these two fragments have not been widely applied in the phylogenetic or phylogeographic study of plant species. This high divergence may be unique to *Z. jujuba* var. *spinosa*. Until now, phylogeographic research concerning sour jujube has yet to be conducted. The distribution centre of sour jujube is located in central China, especially areas around the Loess Plateau, and the present study provides valuable potential molecular markers with sufficient variation information for application in relevant studies.

Contraction and expansion at the boundaries of IR regions are relatively common during plastid evolution, and are hypothesized to explain the variation in size of plastomes (Kim and Lee 2005; Zhu *et al*. 2016). Also, differences in plastome size can be attributed to variability in the length of the SC regions. The large-scale structural features of IR contraction and expansion of plant plastomes were found to be largely conserved across different individuals or lineages. As has been found in other tree genera such as *Pterocarpus* and *Populus* (Hong *et al*. 2020; Zhou *et al*. 2021), junction boundaries of IR regions generally varied within 50 bp. In the present study, it was found that the IR boundaries of all sour jujube individuals showed high conservation. The length of the plastomes of the 21 sour jujube individuals varied by about 2 kb, which mainly resulted from large-scale fragment indels in intergenic regions (Figure 3). Therefore, we cannot discuss the differences in the size of the sour jujube plastomes only from the perspective of the contraction and expansion of the IR regions because the conservative nature of the plastome may cause researchers to ignore other complicated factors (Liu *et al*. 2021). For example, genomic structural changes that occur in intergenic regions may play an additional evolutionary role, but they are difficult to detect because intergenic regions have no coding function and generally show high variation; this may be related to inserted sequence or an intermediate form of plastomic evolution (Liu *et al*. 2021). As the number of published plastomes increases, the evolutionary processes leading to sequence variation should become clearer.

In the present study, a large number of repeat regions and types, including tandem repeat structures and SSRs, were found in sour jujube plastomes, which could be important hotspots for genome reconfiguration (Takayuki *et al*. 2004; Gao *et al*. 2009). In particular, the occurrence of large fragmental repeats, such as the 382 bp palindromic repeat observed in the plastome of individual JZTG11, was speculated to result in an unstable genome structure due to inappropriate rearrangement as repeat sequences provide the potential for genome rearrangement within or between molecules by homologous recombination (Palmer 1985; Yamada 1991; Maréchal and Brisson 2010). As a very powerful type of molecular marker, SSRs are widely used in various studies, due to their high level of polymorphism and cost effectiveness (Wang *et al*. 2009; Bai *et al*. 2010; Zeng *et al*. 2016). CpSSRs and nSSRs for sour jujube have been developed and utilized in population genetics studies (Huang *et al*. 2015; Zhang *et al*. 2015), but the number of SSR markers developed was relatively small compared to the substantial number of SSR loci detected in the present study. Different SSR motifs appeared at different frequencies in the sour jujube plastomes. We found that the predominant mononucleotide repeat in all analyzed plastomes was the A/T motif, which accounted for nearly 100% of the mononucleotide repeats, and only one di- and trinucleotide repeat motif were detected [see Supporting Information—Table S4]. Most studies have shown that the predominant cpSSRs of land plants are consistent with their AT-biased plastomes (Yuan *et al*. 2021; Zhou *et al*. 2021). An exception was found in the plastome of Polypodiaceae, in which the majority of mononucleotide repeats were C/G (Liu *et al*. 2021). This was hypothesized to be one of the molecular foundations contributing to the adaption of Polypodiaceae to the environment. Stress tolerance of sour jujube to various environmental conditions was relatively strong compared to Chinese jujube (Wang *et al*. 2018), and the potential contribution of plastome composition to high stress tolerance needs to be clarified. The cpSSRs scanned in our study provide unique information for investigating genetic structure and genetic variation of sour jujube and these cpSSRs will be complementary and comparable to nSSRs. In addition, these repeats provide many informative loci for the development of molecular markers for future phylogenetic, phylogeographic and population genetics study of sour jujube, and even *Ziziphus* and Rhamnaceae.

Direct selection on functional genes in plant plastomes is relatively rare, indicating that purifying selection maintains functional continuity in chloroplast functional genes (Yoshihiro *et al*. 2002). However, various comparative plastomic studies have revealed that a handful of chloroplast genes evolved via positive or negative selection (Huang *et al*. 2017; Jiang *et al*. 2018; Yu *et al*. 2019). For example, Huang *et al*. (2017) found that *ycf1*, *ccsA*, *rpl16* and *rps12* underwent positive selection within the sour jujube plastome. Our findings also demonstrate that several genes were under positive selection, including *atpE*, *rpl16* and *rps3*. These functional genes belong to different gene groups in the plant plastome [see Supporting Information—Table S3], and their potential roles in the evolution of sour jujube need further study. Discrepancies between our research and Huang *et al*. (2017) except for *rpl16* may result from the differences in analytical materials. In the present study, we calculated the Ka/Ks ratio with 23 sour jujube and another five *Ziziphus* species’ plastomes while Huang *et al*. (2017) only used four plastomes from *Ziziphus* species in their calculation. Results from this research may reveal the intraspeciess’ evolutionary pressure on the sour jujube plastome to a deeper and more precise extent. Given these findings, more detailed and widespread studies need to be conducted to document and improve our understanding of the role of selection in plastome evolution. Studies that integrate gene expression data and evolutionary rate of nuclear-chloroplast-interacting genes may clarify why different chloroplast genes have undergone different modes and rates of selection.

While the plastome is generally viewed as highly conserved and little changed, other findings have suggested that the plastome contains ample SNPs for resolving various issues (Elmosallamy *et al*. 2019). With the accumulation of sequencing data, SNPs have become a powerful and commonly used molecular marker in phylogenomic, population genomic and other relevant research (Huang *et al*. 2016; Wang *et al*. 2020). In the present study, only 80 SNPs were detected in the 21 sour jujube plastomes, with SNPs containing gaps excluded (results not shown). Most SNPs were located in the non-coding region, indicating high variation compared to protein-coding sequences as revealed in mVISTA analysis. The utility of these SNPs in uncovering the population structure of sour jujube populations distributed in China was assessed in another study (Du *et al.* submitted paper). More work is needed to understand how these changes may lead to functional changes between different populations (Zhou *et al*. 2021).

The classification and delimitation of intra-Rhamnaceae lineages are controversial, resulting from the large number of species, highly variable morphology as well as discrepancy between different data types from various studies (Takhtajan 1980; Thorne 1992; Richardson *et al*. 2000a). Richardson *et al*. (2000b) constructed the phylogeny of Rhamnaceae using two plastid fragments and found that the phylogenetic tree supported the monophyly of the family and three strongly supported clades were identified. However, no morphological characters of these three clades were found to underpin a formal taxonomic description, which indicates that morphological and molecular classification are difficult to coordinate in Rhamnaceae. In the phylogenomic tree constructed in the present study, a similar phenomenon was revealed. Traditionally, Rhamnaceae is divided into five tribes, including Rhamneae, Zizipheae, Ventilagineae, Colletieae and Gouanieae, based on morphological characters (Suessenguth 1953), but a molecular phylogeny proposed an 11-tribe classification of this family (Richardson *et al*. 2000b). We found that species from different tribes clustered together in the same branch, especially in Clade II (Figure 6). Although the sample number in this study was relatively small, this may imply that a combination of morphological, nuclear and plastomic data is needed to explicitly explore the phylogeny pattern of Rhamnaceae. Furthermore, it was found in the phylogenetic tree that all the sour jujube and Chinese jujube individuals clustered together in a single branch, in which sour jujube plastomes were clearly sister to the Chinese jujube plastomes occupying some terminal positions. This phylogenetic pattern clearly showed the close relationship between these two lineages and suggested that the classification of these two lineages as a single species would be more convincing. The Latin name, *Ziziphus acidojujuba* C. Y. Cheng et M. J. Liu, of sour jujube may not be appropriate from the perspective of plastome phylogeny. *Z. jujuba* may have originated or been domesticated from *Z. jujuba* var. *spinosa* (Guo *et al*. 2020). Recently, conducted phylogenomic analyses based on whole-genome resequencing of SNP data also led to this conclusion (Huang *et al*. 2016; Guo *et al*. 2021; Shen *et al*. 2021). Although the number of plastomes of Chinese jujube used in the present study was relatively small, we may speculate that the phylogenetic position of sour jujube and Chinese jujube does not alter with the accumulation of data resulting from the established status of sour jujube to Chinese jujube in the various studies mentioned above. Sour jujube is considered as a valuable gene pool, for example for the functional genes underlying high biotic/abiotic stress tolerance and resistance to fruit splitting, and will provide a valuable resource for future genetic improvement of Chinese jujube (Zhang *et al*. 2015; Guo *et al*. 2020).

## Conclusions

In the present study, plastomes of *Z. jujuba* var. *spinosa* were assembled and comparative plastome and phylogenomic analyses were conducted. The conservative nature of the plant plastome was detected in the sour jujube plastomes, but some intriguing variations were also uncovered, such as some highly divergent loci and functional genes under positive selection. The CpSSRs and SNPs identified in sour jujube plastomes provide informative molecular markers for future studies. Phylogenomic analysis indicated the sister relationship of sour jujube to Chinese jujube. The plastome contains sufficient information about variation and has been shown to be effective in plant biology research (Dong *et al*. 2017; Zhao *et al*. 2018). Using the plastome is the ultimate way to assess diversity within a species, allowing for the detection of all kinds of variations, including SNPs, indels and even structural variants (Mishra *et al*. 2021).

## Supplementary Material

plad006_suppl_Supplementary_Table_S1-S5_S7-S9

plad006_suppl_Supplementary_Table_S10

plad006_suppl_Supplementary_Table_S6

## Data Availability

The data presented in this study are openly available in NCBI with accession number ON611607-ON611627.
