# Supplementary material for: A comparative plastomic analysis of Ziziphus jujuba var. spinosa (Bunge) Hu ex H. F. Chow and implication of the origin of Chinese jujube
Source: AoB Plants. 2023 Feb 21;15(2):plad006. doi: 10.1093/aobpla/plad006 (PMC10071050; doi:10.1093/aobpla/plad006)
Supplement: plad006_suppl_Supplementary_Table_S1-S5_S7-S9 [file plad006_suppl_supplementary_table_s1-s5_s7-s9.pdf]

Table S1 Sampled individuals of *Z. jujuba* var. *spinosa* in this study

Number	Province	Population location	Individual code	Geographical information	Herbarium number
1	Hebei	Longhua County, Chengde	HBCD9	41.39 °N 117.85 °E	NDLXHBCD9
2		Haigang District,Qinhuangdao	HBQHD5	40.01 °N 119.52 °E	NDLXHBQHD5
3		Renze District, Xingtai	HBXT5	37.13 °N 114.76 °E	NDLXHBXT5
4	Shanxi	Changzhi County，Changzhi	CZCZ1	35.35 °N 112.56 °E	NDLXCZCZ1
5		Yungang District，Datong	DTYG9	39.61 °N 112.86 °E	NDLXDTYG9
6		Taigu District，Jinzhong	JZTG11	36.91 °N 112.42 °E	NDLXJZTG11
7		Xiaoyi County，Lvliang	LLXY7	36.65 °N 111.78 °E	NDLXLLXY7
8		Ruicheng County，Yuncheng	YCRC1<br>4	34.69 °N 110.69 °E	NDLXYCRC1
9	Shandong	Laoshan District, Qingdao	SDLS10	36.18 °N 120.54 °E	NDLXSDLS10
10		Mengyin County, Linyi	LYMY1<br>9	35.56 °N 118.34 °E	NDLXLYMY1
11		Taishan District, Taian	SDTA10	36.21 °N 117.12 °E	NDLXSDTA10
12	Tianjin	Wuqing District, Tianjin	TJWQ2	39.48 °N 117.02 °E	NDLXTJWQ2
13	Ningxia	Yongning County, Yinchuan	NXYC4	38.18 °N 106.19 °E	NDLXNXYC4
14	Shaanxi	Nanzheng County, Hanzhong	SXHZ9	32.92 °N 109.79 °E	NDLXSXHZ9
15		Linwei District, Weinan	SXWN9	34.39 °N 113.44 °E	NDLXSXWN9
16	Henan	Dengzhou County, Nanyang	HNNY2	32.64 °N 111.93 °E	NDLXHNNY2
17		Lushi County, Samenxia	HNSMX5	34.08 °N 110.94 °E	NDLXHNSMX5
18		Yiyang County, Luoyang	HNLY9	34.49 °N 112.13 °E	NDLXHNLY9
19		Zhongmou County,Zhengzhou	HNZZ1<br>4	34.88 °N 114.03 °E	NDLXHNZZ1
20	Anhui	Shucheng District, Liuan	AHLA1	31.41 °N 117.08 °E	NDLXAHLA1
21	Hubei	Nanzhang County, Xiangyang	HBXY1<br>4	31.71 °N 111.78 °E	NDLXHBXY1

Table S2 Chloroplast genome of previously reported Rhamnaceae species downloaded from NCBI

Tribe	Species	Accession number
Zizipheae	*Z. jujuba* var. *spinosa*	
	voucher Suanzao-007	KX266830
		MW160433
	Z. jujuba	
	cultivar 'Dongzao'	MF781071
	cultivar 'Junzao'	KX266829
		NC030299
	Z. mauritiana	NC037151
	Z. spina-christi	NC037152
	Z. incurva	NC050251
	Z. attopensis	MW201670
	Berchemiella wilsonii	NC043912
	Berchemiella wilsonii var. *wilsonii*	KY926621
	Berchemia lineata	NC051560
	Berchemia flavescens	MK460212
	Berchemia berchemiifolia	NC037477
Ventilagineae	*Ventilago leiocarpa*	NC053785
Pomaderrea	*Spyridium parvifolium* var. *parvifolium*	MH234313
Rhamneae	*Rhamnus taquetii*	NC045855
	Rhamnus heterophylla	MT211599
	Rhamnus globosa	MT360052
	Rhamnus crenata	LC635131
	Hovenia trichocarpa	NC052875
	Hovenia dulcis	MT225403
	Hovenia acerba	MN782301

Table S3 List of predicted functional genes in the sour jujube plastome

Category	Gene group (number)	Gene name
Self replication	Ribosomal RNA genes (4)	*rrn4.5*# *rrn5*# *rrn 16*# *rrn23*#
	Transfer RNA genes (31)	*trnA-UGC#^ trnC-GCA trnC-ACA^ trnD-GUC trnE-UUC#^ trnF-GAA trnfM-CAU trnG-GCC trnG-UCC^ trnH-GUG trnI-CAU# trnI-GAU#^ trnK-UUU^ trnL-CAA# trnL-UAA^ trnL-UAG trnM-CAU trnN-GUU# trnP-UGG trnQ-UUG trnR-ACG# trnR-UCU trnS-GCU trnS-GGA trnS-UGA trnT-CGU^ trnT-GGU trnT-UGU trnV-GAC# trnW-CCA trnY-GUA*
	Small subunit of ribosome (12)	*rps2 rps3 rps4 rps7# rps8 rps11 rps12#^ rps14 rps15 rps16^ rps18 rps19#*
	Large subunit of ribosome (9)	*rpl2#^ rpl14 rpl16 rpl20 rpl22 rpl23# rpl32 rpl33 rpl36*
	DNA-dependent RNA polymerase (4)	*rpoA rpoB rpoC1^ rpoC2*
	Translational initiation factor (1)	*infA*
Photosynthesis	NADH dehydrogenase (11)	*ndhA^ ndhB#^ ndhC ndhD ndhE ndhF ndhG ndhH ndhI ndhJ ndhK*
	Photosystem I (6)	*psaA psaB psaC psaI psaJ pafI^^*
	Photosystem II (14)	*psbA psbB psbC psbD psbE psbF psbH psbI psbJ psbK psbL psbM psbT psbZ*
	Cytochrome b/f complex (6)	*petA petB petD petG petL petN*
	ATP synthase (6)	*atpA atpB atpE atpF^ atpH*

		atpI
	Large subunit of rubisco (1)	*rbcL*
Other genes	Maturase (1)	*matK*
	Envelope membrane protein (1)	*cemA*
	Subunit of acetyl-CoA (1)	*accD*
	c-type cytochrome synthesis (1)	*ccsA*
	Component of TIC complex (1)	*ycf1*
	Protease (1)	*clpP*^^
	Conserved ORF (2)	*pbfI pafII*
Unknown function	(2)	*ycf2# ycf15#*

Note: # indicated duplication of this predicted functional gene, ^ indicated one intron and ^^ indicated two introns.

Table S4 Primers developed to verify the highly diverged regions and SNPs in sour jujube plastome

Locus	Primers (5’-3’)	Tm (℃)
matK	F: GAACCCCTTCTTCCTGCGTAA	59.51
	R: ATCTCAGCAACACGATCTCCT	60.56
accD	F: TCGATGTTGTTTAGGAGGGGG	60.54
	R: TGTCACCCCACCCGTAGTAG	58.55
ycf2	F: TCGACCGTGACCTTGATACG	59.55
	R: TGCATGGTTCCACTCTGCAA	60.18
rpoB	F: CCACACCTCGCATGAATTGC	60.18
	R: ATGCTTGGGGATGGAACTGA	59.00
ycf1	F: AACCCCGCTTCCGTATTGTT	59.96
	R: TCACAACCCTCAGCCCTTAAC	59.99
139545 bp (A-C)	F: ACAATTACCGCGAGCAAACA	58.77
	R: CACAACCGGCCAAAAAGGG	59.93
126314 bp (T-G)	F: TTTACAACCGACACAGTCCTCT	59.64
	R: GGCTCCCTTACATGAGTCGG	59.14
116018 bp (G-T)	F: AAGGACCTTTCCCTTTGGGG	59.51
	R: GAGGAAGAATTACGGGGCGT	59.82
71959 bp (G-T)	F: GCCTCAAGAGTTGCTCAAGG	58.84
	R: AAAGCACTTCCCTAAGTTTCCA	57.82
66043 bp (A-G)	F: GTGTGGCTTCTTCCGAGCAT	60.67
	R: GTTGCATTTTATTGGAATTGGGGA	58.50
52525 bp (C-G)	F: ATGGGCAATCCTGAGCCAAA	59.96
	R: ATCGATTCACAGCAATTCTTTCAT	57.55
45888 bp (A-G)	F:TGAACCTATGAGAGATGTTTAGACT	57.04
	R: TGACTGTTCACACGGTTTGG	58.63
35886 bp (G-T)	F: TTTTCCCAACGGGATTCAAGA	57.77
	R: CCCTGAACTCAATTCGGTTATCCT	60.38
12602 bp (A-C)	F: TCCAAAAGGCGTGAATGCAA	58.96
	R: GCCGATTCTCCGGCTACATTA	60.00
5769 bp (A-G)	F: TCCGTAAAAACCCCCGCTTT	59.00
	R: GAGCCGTACGAGGAGAAAACT	60.00

Table S5 The number and motif type of SSRs in sour jujube plastome

SSR Type	Repeat unit	Amount																				
		AHLA1	HBCD9	CZCZ1	DTYG9	HBXY14	SXHZ9	JZTG11	LLXY7	HNLY9	LYMY19	NXYC4	HNNY2	SDLS10	HBQHD5	HNSMX5	SDTA10	TJWQ2	SXWN9	HBXT5	YCRC14	HNZZ14
Mono	A/T	61	62	63	63	63	65	66	61	60	67	67	60	63	63	62	64	64	66	62	63	64
-	C/G		1	1						1		1				1	1	1				1
Di-	AT/TA	3	2	3	2	4	3	9	2	2	6	3	2	4	9	3	5	4	2	3	4	4
Tri-	AAT/ATT		1	1		1	1	1			1	2		1	1		1	1	1	1	1	2
Total		64	66	68	65	68	69	76	63	63	74	73	62	68	73	66	71	70	69	66	68	71

Table S7 The long repeats detected in the sour jujube plastomes

Individual	Type	Length				Summary
		30-39 bp	40-49 bp	>=50 bp	Total	
AHLA1	F	25	0	6	31	
	P	25	1	5	31	
	R	3	0	0	3	
	C	0	0	0	0	65
HBCD9	F	28	0	13	41	
	P	27	1	7	35	
	R	2	0	0	2	
	C	0	0	0	0	78
CZCZ1	F	27	1	9	37	
	P	29	1	9	39	
	R	3	0	0	3	
	C	1	0	0	1	80
DTYG9	F	27	0	9	36	
	P	24	3	4	31	
	R	1	0	0	1	
	C	0	0	0	0	68
HBXY14	F	28	0	8	36	
	P	30	0	6	36	
	R	5	0	0	5	
	C	1	0	0	1	78
SXHZ9	F	28	0	11	39	
	P	31	0	9	40	
	R	5	0	0	5	
	C	1	0	0	1	85
JZTG11	F	26	3	3	32	
	P	31	4	8	43	
	R	6	0	0	6	
	C	4	0	0	4	85
LLXY7	F	26	0	9	35	
	P	24	2	13	39	
	R	3	0	0	3	
	C	0	0	0	0	77
HNLY9	F	27	0	9	36	
	P	25	0	11	36	
	R	3	0	0	3	
	C	1	0	0	1	76
LYMY19	F	26	3	2	31	
	P	23	4	0	27	
	R	2	3	0	5	
	C	0	1	0	1	64
NXYC4	F	26	0	8	34	

	P	31	2	4	37	
	R	2	0	0	2	
	C	0	0	0	0	73
HNNY2	F	27	2	9	38	
	P	29	0	8	37	
	R	3	0	0	3	
	C	1	0	0	1	79
SDLS10	F	28	0	9	37	
	P	31	2	4	37	
	R	2	0	0	2	
	C	0	0	0	0	76
HBQHD5	F	20	2	2	24	
	P	30	2	4	36	
	R	0	1	0	1	
	C	0	0	0	0	61
HNSMX5	F	27	0	7	34	
	P	25	0	11	36	
	R	6	0	0	6	
	C	1	0	0	1	77
SDTA10	F	28	0	10	38	
	P	31	2	4	37	
	R	2	0	0	2	
	C	0	0	0	0	77
TJWQ2	F	27	2	1	30	
	P	32	2	1	35	
	R	3	0	0	3	
	C	1	0	0	1	69
SXWN9	F	29	1	5	35	
	P	27	2	2	31	
	R	2	0	0	2	
	C	0	0	0	0	68
HBXT5	F	30	0	9	39	
	P	29	2	5	36	
	R	4	0	0	4	
	C	1	0	0	1	80
YCRC14	F	29	1	8	38	
	P	29	2	4	35	
	R	2	0	0	2	
	C	0	0	0	0	75
HNZZ14	F	27	2	1	30	
	P	31	2	1	34	
	R	3	0	0	3	
	C	1	0	0	1	68

Note: F, P, R and C indicates forward, palindromic, reverse and complementary repeats.

Table S8 Ka/Ks value of 80 protein coding genes in sour jujube plastomes

Protein coding gene	Ks	Ka
accd	0.0029	0.0014
atpA	0.0107	0.0023
atpB	0.0062	0.0015
atpE[*]	0.0014	0.0016
atpF	0.0223	0.0201
atpH	0.0010	0
atpI	0.0059	0.0014
ccsA	0.0094	0.0030
cemA	0.0095	0.0004
clpP1	0.0014	0.0004
matK	0	0
ndhA	0.0082	0.0016
ndhB	0.0004	0.0001
ndhC	0.0082	0.0005
ndhD	0.0038	0.0007
ndhF	0.01	0.0023
ndhG	0.0072	0.002
ndhH	0.0039	0.0009
ndhI	0.0088	0.0003
ndhJ	0.0035	0.0002
ndhK	0.0087	0.0014
petA	0.0052	0.0021
petB	0.0030	0.0014
petD	0.0070	0.0019
petG	0.0022	0.0008
petL	0	0.0010
petN	0.0028	0
psaA	0.0083	0.0005
psaB	0.0046	0.0004
psaC	0.0034	0.0014
psaI	0.0052	0.0005
psaJ	0.0058	0.0014
psbA	0.0050	0.0018
psbB	0.0052	0.0009
psbC	0.0055	0.0027
psbD	0.0036	0.0010
psbE	0.1316	0.0522
psbF	0.0097	0.0015
psbH	0	0.0016
psbI	0.0148	0
psbJ	0	0
psbK	0.0091	0.0044

psbL	0	0.0731
psbM	0.0049	0
psbT	0	0.0008
psbZ	0	0.0005
rbcL	0.0097	0.0021
rpl2	0.0011	0
rpl14	0	0.0006
rpl16[*]	0.0070	0.0087
rpl20	0.0029	0.0025
rpl22	0.0043	0.0020
rpl23	0	0
rpl32	0.0083	0.0021
rpl33	0.0027	0.0018
rpl36	0	0.0146
rpoA	0.0101	0.0035
rpoB	0.0084	0.0028
rpoC1	0.0028	0.0012
rpoC2	0.0069	0.0029
rps3[*]	0.0014	0.0021
rps4	0	0.0009
rps7	0.0006	0
rps8	0.0033	0.0019
rps11	0.0066	0.0033
rps12	0.0010	0
rps14	0.0087	0.0006
rps15	0.0117	0.0043
rps16	0.0236	0.0069
rps18	0.0036	0.0006
rps19	0.0029	0.0018
ycf1	0	0.1006
ycf2	0.0004	0.0003

Table S9 SNP location and variation pattern in the sour jujube plastomes

Gene	Name	Location	Variant Nucleotide	Variant Frequency
ycf2	A	160974	A	66.70%
	T	160974	T	33.30%
CNS	A	139984	A	42.90%
	T	139984	T	57.10%
CNS	A	139545	A	33.30%
	C	139545	C	66.70%
ycf1	G	137119	G	52.40%
	T	137119	T	47.60%
ycf1	G	134171	G	38.10%
	T	134171	T	61.90%
ycf1	C	134147	C	33.30%
	T	134147	T	66.70%
ycf1	G	133832	G	33.30%
	T	133832	T	66.70%
rps15	A	132384	A	47.60%
	G	132384	G	52.40%
ndhA	G	130487	G	47.60%
	T	130487	T	52.40%
ndhI	G	128418	G	42.90%
	T	128418	T	57.10%
CNS	C	126841	C	38.10%
	G	126841	G	61.90%
CNS	G	126314	G	33.30%
	T	126314	T	66.70%
ndhD	C	124640	C	33.30%
	T	124640	T	66.70%
CNS	A	124183	A	52.40%
	T	124183	T	47.60%
CNS	A	124182	A	52.40%
	C	124182	C	47.60%
CNS	G	124179	G	38.10%
	T	124179	T	61.90%
CNS	A	124178	A	61.90%
	T	124178	T	38.10%
CNS	A	124177	A	38.10%
	C	124177	C	61.90%
CNS	G	124174	G	52.40%
	T	124174	T	47.60%
CNS	A	124173	A	52.40%
	T	124173	T	47.60%
ccsA	A	123445	A	57.10%
	T	123445	T	42.90%

CNS	A	122332	A	66.70%
	G	122332	G	33.30%
CNS	G	121493	G	33.30%
	T	121493	T	66.70%
CNS	G	116018	G	66.70%
	T	116018	T	33.30%
CNS	A	115580	A	47.60%
	T	115580	T	52.40%
ycf2	A	94573	A	33.30%
	T	94573	T	66.70%
rps3	A	90411	A	57.10%
	C	90411	C	42.90%
rps3	G	90387	G	42.90%
	T	90387	T	57.10%
rpoA	G	85452	G	42.90%
	T	85452	T	57.10%
rpoA	G	85006	G	42.90%
	T	85006	T	57.10%
CNS	A	84853	A	38.10%
	G	84853	G	61.90%
CNS	A	84852	A	61.90%
	G	84852	G	38.10%
CNS	A	84071	A	66.70%
	C	84071	C	33.30%
psbB	C	79867	C	61.90%
	T	79867	T	38.10%
CNS	A	73918	A	38.10%
	C	73918	C	61.90%
CNS	C	73013	C	71.40%
	T	73013	T	28.60%
CNS	A	72245	A	33.30%
	C	72245	C	66.70%
CNS	G	71959	G	71.40%
	T	71959	T	28.60%
CNS	A	71649	A	38.10%
	G	71649	G	61.90%
CNS	A	66043	A	33.30%
	G	66043	G	66.70%
CNS	A	66006	A	28.60%
	G	66006	G	71.40%
CNS	A	65974	A	28.60%
	G	65974	G	71.40%
accD	A	63853	A	28.60%
	G	63853	G	71.40%

rbcL	A	61660	A	52.40%
	C	61660	C	47.60%
CNS	G	60554	G	71.40%
	T	60554	T	28.60%
CNS	A	60246	A	71.40%
	G	60246	G	28.60%
CNS	C	60176	C	66.70%
	T	60176	T	33.30%
CNS	A	60107	A	71.40%
	C	60107	C	28.60%
intron *trnC-ACA*	G	57298	G	52.40%
	T	57298	T	47.60%
CNS	A	53779	A	42.90%
	G	53779	G	57.10%
intron *trnL-UAA*	C	52525	C	42.90%
	G	52525	G	57.10%
CNS	A	45888	A	33.30%
	G	45888	G	66.70%
psaA	C	43423	C	61.90%
	T	43423	T	38.10%
CNS	C	40393	C	47.60%
	T	40393	T	52.40%
CNS	A	39774	A	71.40%
	T	39774	T	28.60%
CNS	G	35886	G	33.30%
	T	35886	T	66.70%
CNS	A	35514	A	28.60%
	C	35514	C	71.40%
CNS	G	35426	G	33.30%
	T	35426	T	66.70%
CNS	A	34315	A	52.40%
	C	34315	C	47.60%
CNS	C	33028	C	52.40%
	G	33028	G	47.60%
CNS	G	32206	G	52.40%
	T	32206	T	47.60%
CNS	G	31120	G	52.40%
	T	31120	T	47.60%
rpoB	A	27498	A	47.60%
	C	27498	C	52.40%
rpoC2	A	21543	A	52.40%
	C	21543	C	47.60%
rpoC2	G	21200	G	47.60%
	T	21200	T	52.40%

rpoC2	G	21000	G	52.40%
	T	21000	T	47.60%
rpoC2	C	19903	C	57.10%
	T	19903	T	42.90%
CNS	C	18361	C	38.10%
	T	18361	T	61.90%
CNS	A	18360	A	38.10%
	T	18360	T	61.90%
CNS	A	18359	A	42.90%
	G	18359	G	57.10%
atpF	G	14295	G	42.90%
	T	14295	T	38.10%
atpF	G	14255	G	57.10%
	T	14255	T	42.90%
atpF	C	14223	C	33.30%
	T	14223	T	66.70%
atpA	A	12602	A	57.10%
	C	12602	C	42.90%
atpA	C	12254	C	57.10%
	T	12254	T	42.90%
rps16	A	6574	A	61.90%
	C	6574	C	38.10%
rps16	A	6573	A	47.60%
	C	6573	C	52.40%
rps16	A	5769	A	57.10%
	G	5769	G	42.90%
CNS	G	5550	G	42.90%
	T	5550	T	57.10%
intron *trnK-UUU*	C	2578	C	47.60%
	G	2578	G	52.40%

Note: CNS indicated noncoding sequences.
